# Trends and patterns in the use of computed tomography in children and young adults in Catalonia — results from the EPI-CT study

**DOI:** 10.1007/s00247-015-3434-5

**Published:** 2015-08-15

**Authors:** Magda Bosch de Basea, Jane A. Salotti, Mark S. Pearce, Jordi Muchart, Luis Riera, Ignasi Barber, Salvador Pedraza, Marina Pardina, Antoni Capdevila, Ana Espinosa, Elisabeth Cardis

**Affiliations:** Centre for Research in Environmental Epidemiology (CREAL), Dr. Aiguader, 88, 08003 Barcelona, Spain; Universitat Pompeu Fabra (UPF), Barcelona, Spain; CIBER Epidemiología y Salud Pública (CIBERESP), Barcelona, Spain; Institute of Health & Society, Newcastle University, Sir James Spence Institute of Child Health, Royal Victoria Infirmary, Newcastle upon Tyne, UK; Hospital Sant Joan de Déu Barcelona, Barcelona, Spain; Corporació Sanitària Parc Taulí, Sabadell, Spain; Hospital Universitari Vall d’Hebron, Barcelona, Spain; Institut de Diagnòstic per la Imatge (IDI), Hospital Universitari de Girona Doctor Josep Trueta, Girona, Spain; Institut d’Investigació Biomèdica de Girona Dr. Josep Trueta (IDIBGI), Girona, Spain; Universitat de Girona, Girona, Spain; Hospital Universitari Arnau de Vilanova, Lleida, Spain; Hospital de la Santa Creu i Sant Pau, Barcelona, Spain; Hospital del Mar Medical Research Institute (IMIM), Barcelona, Spain

**Keywords:** Adolescents, Children, Cohort study, Computed tomography, Epidemiology, Trends

## Abstract

**Background:**

Although there are undeniable diagnostic benefits of CT scanning, its increasing use in paediatric radiology has become a topic of concern regarding patient radioprotection.

**Objective:**

To assess the rate of CT scanning in Catalonia, Spain, among patients younger than 21 years old at the scan time.

**Materials and methods:**

This is a sub-study of a larger international cohort study (EPI-CT, the International pediatric CT scan study). Data were retrieved from the radiological information systems (RIS) of eight hospitals in Catalonia since the implementation of digital registration (between 1991 and 2010) until 2013.

**Results:**

The absolute number of CT scans annually increased 4.5% between 1991 and 2013, which was less accentuated when RIS was implemented in most hospitals. Because the population attending the hospitals also increased, however, the rate of scanned patients changed little (8.3 to 9.4 per 1,000 population). The proportions of patients with more than one CT and more than three CTs showed a 1.51- and 2.7-fold increase, respectively, over the 23 years.

**Conclusion:**

Gradual increases in numbers of examinations and scanned patients were observed in Catalonia, potentially explained by new CT scanning indications and increases in the availability of scanners, the number of scans per patient and the size of the attended population.

## Introduction

Computed tomographic scanning is an extremely informative diagnostic technique, with a wide range of clinical applications. Because of the highly reduced imaging time compared to other techniques such as MRI, CT scanning suits all age groups, including children, without the need for anaesthesia or sedation. Since the introduction of the first CT unit in 1970, both the use of CT scans and the knowledge base and concerns about the potential deleterious health effects of ionising radiation exposure have grown in parallel. Over the last 20 years, CT scanner availability has rapidly increased in most European countries [1], including Spain, where the number of CT scan units per 100,000 population nearly doubled between 1996 and 2011 [2].

In Spain, almost 4 million scans were performed in 2011, representing a rate of 85 CT scans per 1,000 people [2], whereas in Greece and the United States, countries with the highest rates of CT scans, the rate was three times higher (320.4 per 1,000 in Greece and 273.8 per 1,000 in the United States) [1].

Although CT scans comprise a small proportion of all diagnostic radiologic procedures, in countries like the United States they contribute nearly half the population’s collective radiation dose from all medical X-ray examinations [3] and in the United Kingdom they account for up to 68% of the population’s collective radiation dose [4]. In the U.S. more than 70 million CT scans are performed every year [5], whereas in the UK it surpasses the 5 million CT scans per year, with an increasing annual rate of 10% [6]. Overall, in Europe CT scanning is a critical source of ionising radiation exposure and its use is expected to increase in the next years with the widespread application of modern multi-slice techniques [7].

Predictions of the potential health impact of CT scanning [8] using a linear-no-threshold dose-response model (which postulates that the risk is linearly related to dose, with no safety threshold) have been controversial in the radiologic community. Some scientists have postulated that CT radiation doses are too low to produce any health effect [9]. However, recent direct assessment of the health effects of paediatric CT scanning through observational studies suggests an increased risk of leukaemia and brain tumours related to multiple CT scan exposure in children and adolescents [10, 11]. Exposure in childhood is known to entail a higher risk of radiation-induced health effects than exposure later in life because childhood exposure is linked to greater cell division in growing and developing tissues and a longer life expectancy for diseases to develop [9, 12, 13]. Therefore the potential adverse health effects of paediatric CT have become an important topic of concern in radiologic protection [13]. Indeed, prediction models suggest that up to 2% of all future neoplasms in the general population of the United States could be attributable to CT scanning [14]. Even though for an individual child, the health risks from one CT scan are likely to be small and the individual benefit may outweigh the risks, many children are likely to receive multiple scans. It is therefore important that paediatric CT scans are properly indicated and performed and that the resulting radiation doses are closely monitored.

Information on the intensity and patterns of use of diagnostic radiology techniques in Spain is sparse. However, within Catalonia (a Northeast autonomous community of Spain) approximately 7.3 million diagnostic examinations were performed in 2007 on a population of 7.2 million people; 83.8% of these diagnostic examinations involved ionising radiation and 7% (532,312) were CT scans [15]. Among the most common indications for CT imaging in children and young adults up to 20 years old are skull trauma and cephalgia with aggravated criteria, general abdominal problems when all other investigations (e.g., ultrasound) are inconclusive, and the diagnosis and follow-up of neoplasms [16].

Principles of radiation protection for medical exposures urge periodic evaluation of CT scan usage and substitution by other diagnostic techniques not involving ionising radiation, when possible, warranting accurate diagnosis without compromising the health of the patient. Monitoring of CT usage is important for updating the appropriateness criteria for ionising radiation imaging. The assessment of the use of CT scanning over time and characterisation of the patients referred for CT scanning (as part of a comprehensive strategy of evaluating individual risk) is a principal goal of the EPI-CT study.

The EPI-CT study (Epidemiological study to quantify risks for paediatric computerized tomography and to optimise doses) is a multinational cohort study of more than 1 million children, adolescents and young adults referred for CT scan supported by the European Union [17]. This paper assesses the patterns of use of CT in the Spanish autonomous region of Catalonia, one of the main contributors to the Spanish component of EPI-CT, among patients younger than 21 years old at the time that they had their first CT scan.

## Materials and methods

### Study population

Catalonia is the second largest autonomous community of Spain, with an estimated 2013 population of 7,553,650 people [18], including 1,599,195 (21.2%) younger than 21 years [19], heterogeneously distributed over the 32,114 km^2^ that constitutes its four provinces (Barcelona, Tarragona, Lleida and Girona).

This study includes 8 public and autonomic-subsidised private hospitals out of 60 in Catalonia that provide health care free at the point of use for all children and young adults [20]. The eight hospitals providing data are among the larger medical centres performing radiology services to the majority of the paediatric and young adult patients in Catalonia (Hospital Sant Joan de Déu Barcelona, Hospital Universitari Vall d’Hebron, Corporació Sanitària Parc Taulí, Hospital Clínic de Barcelona, Hospital de la Santa Creu i Sant Pau, Hospital Universitari Joan XXIII de Tarragona, Hospital Universitari de Girona Dr. Josep Trueta and Hospital Universitari Arnau de Vilanova). These hospitals are part of the Catalan Health Care System (Xarxa Hospitalària d’Utilització Pública–Departament de Salut) and have been funded from autonomic general taxation since 2001. It is reported that 24.3% of the Catalan adult population supplements their medical coverage through private health care, whereas only 17% of the population younger than 15 years uses private medical services [21]. No private hospitals were included in the study. Ethical approval was gained at each of the participating hospitals, as well as at the coordinating centre, Parc de Salut Mar, to retrospectively access these data.

Patients included in the study were accrued from the records of the radiology departments and had at least one CT scan before the age of 21 years. Completeness of data, defined as the collection of radiological information system (RIS) data from the start of CT scanning at the hospital, was only attained at one hospital. At the others, early records were not computerised and it was not possible to do so in the framework of the project.

### CT scan data

The radiologic data were retrieved from the RIS (Radiology Information System) in each participating hospital since RIS implementation (which occurred heterogeneously between 1991 and 2010 among the hospitals) until December 2013. It is worth noting that by 2004 RIS was fully operating in all except for one small hospital, which in the following years only accounts for 1.6% of all scans in the study. RIS is a computerized system used to store, handle and circulate patient radiologic data. The RIS includes both patient identifying information and basic variables about the examination (including body part scanned and examination date and, in certain instances, indication for the CT scan and the referring hospital department). At one hospital, 2009–2011 data were extracted from the PACS (picture archiving and communication system) and SAP software (SAP SE, Walldorf, Germany) instead of RIS because the hospital discontinued RIS use and migrated to a more complex radiologic management system.

Data were obtained within the Spanish part of the multinational EPI-CT long-term cohort study.

Examination descriptions were grouped into six categories based on body part scanned, using Pearce et al.’s [22] adaptation of the categories defined by Mettler and collaborators [23]. This was done to ensure comparability of scan type groupings with the other EPI-CT participating countries. The categories were: head (including the neck), thorax, abdomen/pelvis, spine, extremities, and “several parts” (a composite of several scan locations in a single examination, e.g., head and thorax, thorax and abdomen, and thorax and pelvis). One of the biggest hospital contributors in terms of patients historically did not record the department ordering the CT examination, so in scans from that hospital the CT ordering service appears as “radiology and nuclear medicine,” the default option.

### Statistical methods

The use of CT imaging in the population younger than 21 years attending the participating hospitals was described using simple descriptive statistics. The data are described by gender and age at the time of the examination. For some analyses, children were categorized according to their age at the time of the scan in categories that ensure comparability with similar studies (0, 1 to <5, 5 to <10, 10 to <15, 15 to <20, and 20 to <21 years). The same reasoning was applied to the categorization of the number of CT scans (1, 2–5, 6–10, 11–20, >20) for descriptive purposes. Chi-square test for homogeneity was used to assess the relationship between categorical variables. Both the number of CT scans and the number of scanned patients from 1991 to 2013 were modelled on the whole data set (including “hospital” as a cluster variable to take into account a random effect related to each hospital) as well as by hospital, fitting a linear regression model using a robust estimator of variance. For this, the outcome variable of these models (number of CT scans and number of scanned patients) was logged and transformed into a normally distributed variable. A generalised linear model with a logit link and binomial distribution was used to individually assess the change in the relative frequency of subjects with one CT scan per year over the study period. The same analysis was conducted to evaluate changes in the relative frequency of subjects with two, three and more than three CT scans per year. Each model was fit to the whole dataset (including hospital as a cluster variable) using a robust estimator of variance (Huber/White sandwich estimator). A similar approach was used to model the variation on the frequency of body part scanned over time. To calculate the rates of CT scans, demographic data from the population served by the participating hospitals were obtained from the General Directorate of Planning and Research on Health (Direcció General de Planificació i Recerca en Salut); these data were available from the Catalan Health Department from 2005 to 2013 by 3-year time-bands. To estimate the population for the 2 years of unavailable data, a regression line was fitted using the official figures provided from 2005 till 2013. No population data were available for earlier periods. The Kruskal–Wallis test was used to compare the rate of CT scans per 1,000 population over time (2005–2013), which followed a non-parametrical distribution. All statistical analyses were performed using Stata (version 12.0; StataCorp, College Station, TX) and statistical significance was set at 0.05.

## Results

**Fig. 1:**
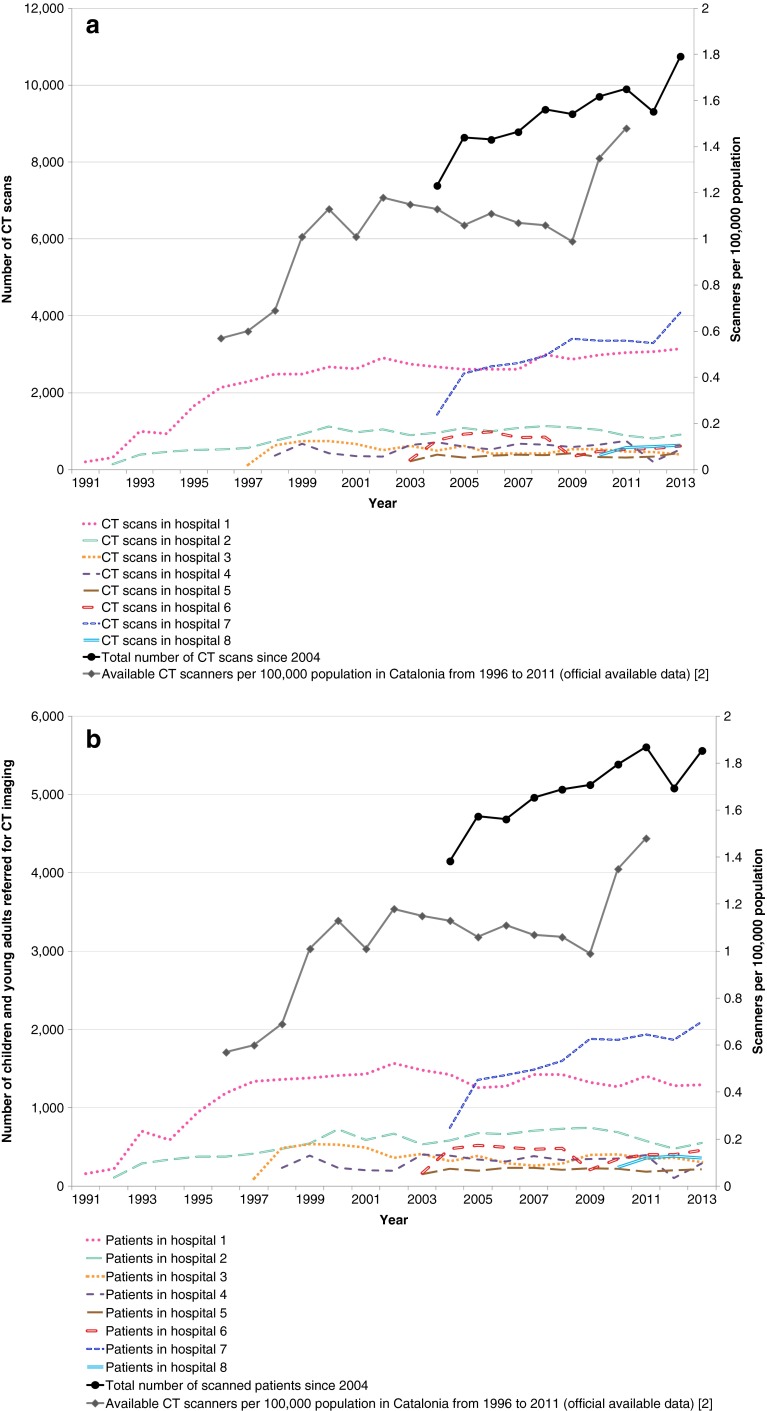
Trends in CT examinations from 1991 to 2013. **a** CT scans per year in children and young adults in Catalonia by hospital. **b** Number of children and young adults referred for CT imaging in Catalonia by hospital

Between 1991 and 2013, 131,655 CT scans were performed on 74,437 children and adults younger than 21 years; 76,543 examinations (58.1%) were performed on 42,238 males (56.7%) and 55,112 (41.9%) examinations on 32,199 females (43.3%) (Table 1). Although the trend in the number of CT scans was heterogeneous among the hospitals, overall the number of CT scans increased by 4.5% (95% confidence interval [CI] 2.0–7.2%) per year between 1991 and 2013. Increasing trends were seen in the number of CT scans in six of the eight participating hospitals, from the implementation of RIS in each hospital until the end of data collection in 2013 (Fig. 1); increases were statistically significant for the two biggest hospitals out of the eight participating centres. These two hospitals, which in 2013 performed 3,200 and 4,100 CT scans, respectively, in patients age 0–20, were among those where CT scan frequency showed a statistically significant anual growth rate of 8.1% and 5.6%, respectively, over the study period (1991 to 2013).

**Table 1:** Characteristics of Catalan children and young adults referred for a CT scan by gender (*Χ* ^2^, *P* < 0.001 for all four categorical variables versus gender)

Categorical variables	Totals	Male	Female
		*n* (%)	*n* (%)
Age at the time of the first scan (years)	74,437 patients	42,238	32,199
< 1	6,796	3,876 (9.2)	2,920 (9.1)
1 to <5	12,807	7,411 (17.5)	5,396 (16.8)
5 to <10	12,911	7,138 (16.9)	5,773 (17.9)
10 to <15	16,003	8,938 (21.2)	7,065 (21.9)
15 to <20	21,807	12,649 (29.9)	9,158 (28.4)
20 to <21	4,113	2,226 (5.3)	1,887 (5.9)
Age at the time of the scan (years)	131,655 CT scans		
< 1	10,555	6,084 (7.9)	4,471 (8.1)
1 to <5	22,573	13,053(17.1)	9,520 (17.3)
5 to <10	23,220	13,107(17.1)	10,113 (18.3)
10 to <15	28,057	15,941 (20.8)	12,116 (22.0)
15 to <20	39,822	24,018 (31.4)	15,804 (28.7)
20 to <21	7,428	4,340 (5.7)	3,088 (5.6)
Body part scanned	131,655 CT scans		
Head and neck	79,885	46,983 (61.4)	32,902 (59.7)
Chest/thorax	23,900	13,520 (17.7)	10,380 (18.8)
Abdomen/pelvis	13,994	8,025 (10.5)	5,969 (10.8)
Spine	5,849	3,456 (4.5)	2,393 (4.3)
Extremities	4,894	2,748 (3.6)	2,146 (3.9)
Several parts	1,338	740 (1.0)	598 (1.1)
Unknown	1,795	1,071 (1.4)	724 (1.3)
Number of CT scans	74,437 patients		
1	52,498	29,178 (69.1)	23,320 (72.4)
2–5	18,797	11,144 (26.4)	7,653 (23.8)
6–10	2,322	1,437 (3.4)	885 (2.7)
11–20	728	435 (1.0)	293 (0.9)
> 20	92	44 (0.1)	48 (0.1)

The overall upward trend in number of CT scans per year declined in 2012 but seemed to recover by the end of the study period (2013). The increasing trend was less pronounced (3.6% per year; 95% CI 1.2–6.0%) but was still statistically significant when considering the number of patients referred for CT scans (Fig. 1). By 2004 seven out of the eight participating hospitals had digitalised their radiology service data and there was a clear upward trend in absolute number of CT scans (Fig. 1) and in absolute number of patients (Fig. 1) over time, driven mainly by the increase in one of the biggest hospitals. The number of CT scanners per 100,000 population in Catalonia also increased during the period of 1996 to 2011 (Fig. 1). Although the starting point in terms of the number of patients and CT scans is similar for male and female patients, substantially more male patients received CT scans through the 23-year period (Table 1).

Median age at the time of the scan was 12.1 years old for male patients (interquartile range [IR]: 5.0–17.1) and 11.6 for female patients (IR: 4.9–16.7) (data not shown); 9.2% of the patients in the study had their first CT scan during the first year of life. The age group that accounted for the largest number of CT scans was the 15- to <20-years age group, with more than 30% of all the examinations performed in this age range. The overall distribution of CT scans by age-bands was very similar to the CT scan distribution in age-bands at the time of the first CT scan. Most patients, 70.5%, received only one CT scan during the study period; 72.4% of all female patients had one CT scan and 23.8% had two to five CTs, while 69.1% of all male patients had one CT scan and 26.4% had two to five CTs (Table 1).

**Fig. 2:**
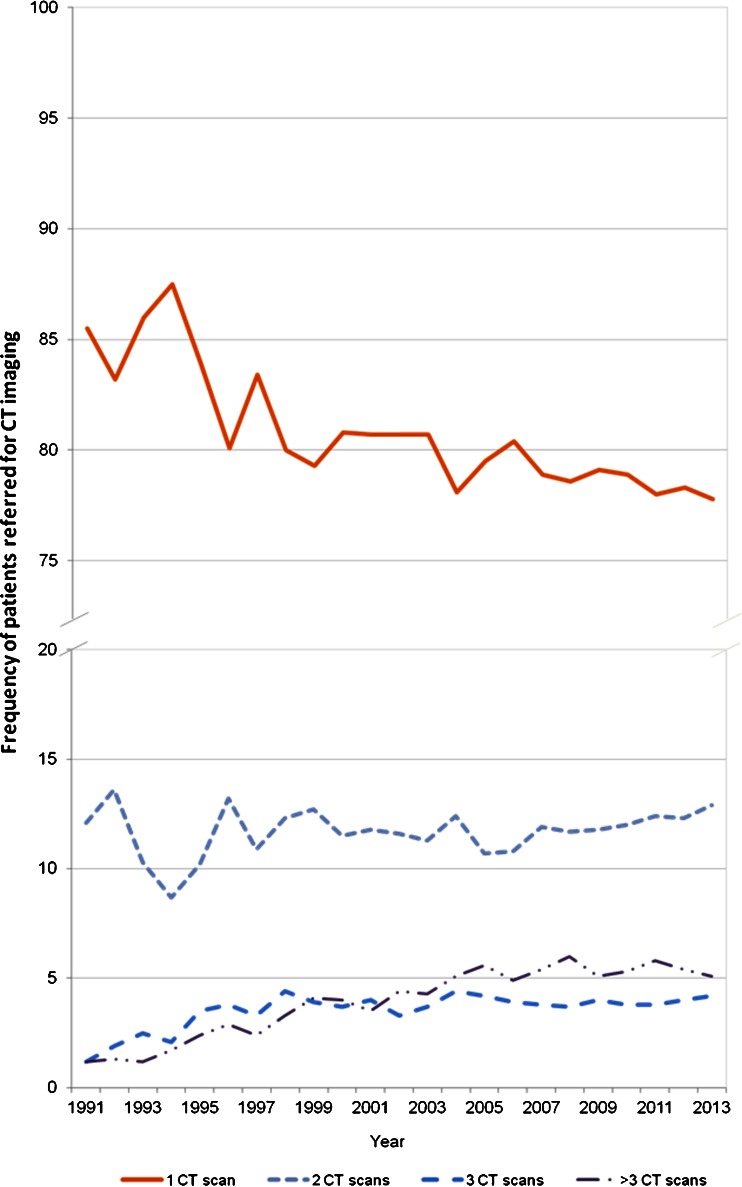
Frequency of patients who received 1, 2, 3 and more than 3 CT scans per year between 1991 and 2013

Within the participating hospitals, the number of CT scans performed in the same patient per year appeared to increase over time (Fig. 2). In 1991, 12% of all the scanned patients received two CT scans, 1.2% received three CT scans and 1.2% received more than three CT scans. In 2013 at the end of data collection, 12.9% of all scanned patients received two CT scans, 4.2% received three CT scans and 5.1% received more than three CT scans; the last category showed the greatest difference between the relative frequency in 1991 and 2013. The percentage of patients requiring only one CT scan showed a 0.6-fold decline between 1991 and 2013 (*P* = 0.076) (Fig. 2). In total, from 1991 to 2013 we observed a non-statistically significant 1.5-fold increase in the relative frequency of patients who received more than one CT scan per year (*P* = 0.076). During the same period the frequency of patients receiving more than three CT scans per year showed a 2.7-fold increase (*P* < 0.001), although from 2004 onwards the trend is almost flat. When analysing by gender, similar trends were observed for male and female patients (data not shown), although comparatively during the total study period the frequency of female patients receiving one CT scan was higher than in males.

A total of 710 examination descriptions were identified from among 131,655 datasets in the study and were grouped into Mettler categories. In 101 examination records, the description of the body part scanned was missing, and 1,795 examination descriptions did not fit into any of the six Mettler categories. Three anatomical regions accounted for 90% of CT scans, with head/neck, thorax, and abdomen/pelvis representing 60.7%, 18.2% and 10.6%, respectively, of all CT scans. No differences were observed by gender (Table 1). The relative frequency of head/neck and abdomen/pelvis scans showed a 0.7-fold and 0.9-fold decrease, respectively, over the 23-year period of the study, whereas the frequency of thorax examinations showed a 1.1-fold increase (data not shown). When the heterogeneity among hospitals was taken into account no statistically significant trend was observed for the three most prevalent body parts scanned.

The distribution of CT scans by anatomical region varied depending on the age of the patient at the time of the diagnostic procedure. For patients younger than 10 years, approximately 66.5% of all CT scans performed were of the head/neck, with 21% of the thorax and 6.8% of the abdomen/pelvis. On the other hand, for patients older than 15 years, approximately 53.6% of CT scans were of the head/neck, 15.6% were of the thorax and 15.9% were of the abdomen/pelvis (Table 2).

**Table 2:** Body part scanned by age range for children and young adults in Catalonia from 1991 to 2013 (*Χ* ^2^, *P* < 0.001)

	Age at the time of the scan (years)					
Body part scanned	<1	1 to <5	5 to <10	10 to <15	15 to <20	20 to <21
	*n* (%)	*n* (%)	*n* (%)	*n* (%)	*n* (%)	*n* (%)
Head/neck	7,286 (69.0%)	15,023 (66.6%)	15,155 (65.3%)	17,154 (61.1%)	21,500 (54.0%)	3,767 (50.7%)
Thorax	2,381 (22.6%)	4,876 (21.6%)	4,580 (19.7%)	4,722 (16.8%)	6,115 (15.4%)	1,226 (16.5%)
Abdomen/pelvis	552 (5.2%)	1,548 (6.9%)	1,769 (7.6%)	2,583 (9.2%)	6,133 (15.4%)	1,409 (19.0%)
Spine	136 (1.3%)	512 (2.3%)	707 (3.0%)	1,349 (4.8%)	2,675 (6.7%)	470 (6.3%)
Extremities	71 (0.7%)	178 (0.8%)	594 (2.6%)	1,630 (5.8%)	2,146 (5.4%)	275 (3.7%)
Several parts	67 (0.6%)	248 (1.1%)	194 (0.8%)	244 (0.9%)	463 (1.2%)	122 (1.6%)
Unknown	62 (0.6%)	188 (0.8%)	221 (1.0%)	375 (1.3%)	790 (2.0%)	159 (2.1%)
Total	10,555 (8.0%)	22,573 (17.1%)	23,220 (17.6%)	28,057 (21.3%)	39,822 (30.2%)	7,428 (5.6%)

Information about the clinical department that requested the CT scan procedure was obtained directly from hospital RIS data for 111,995 CT scans (85.1% of all CT scans collected). Paediatrics departments requested 15.4% of the scans, followed by the surgery (9.2%), neurosurgery and neurology (8.6%), emergency (7.1%) and oncology and haematological malignancies (5.2%) (Table 3). There were statistically significant differences between referring specialities when comparing male with female patients (excluding “obstetrics and gynaecology” from the comparison) (*P* < 0.001). Approximately 12.2% of all CT scans performed in patients younger than 5 years were requested by neurosurgery and neurology, and this frequency decreased to 5.5% for the 20-year-old patients. On the other hand, the emergency department requested 4.1% of all CT scans in those younger than 15 years and requested up to 13.1% of all CT scans performed in patients age 15 to <20 years. As might be expected, the frequency of the scans requested by paediatric departments decreased with the age of the patients. As explained in methods, in one of the biggest participating hospitals the “radiology and nuclear medicine” department was the default option when recording the department ordering the CT scan, which is not very informative of the CT scan distribution among medical departments in that larger hospital (Table 3).

**Table 3:** Absolute and relative frequency of CT scans referred by medical specialty for total individuals and by gender and age in six categories (*Χ* ^2^, *P* < 0.001 for both genders and age groups)

	CT scans per referring department								
	Total	Gender		Age (years)					
		Males	Female	<1	1 to <5	5 to <10	10 to <15	15 to <20	20 to <21
Referrer	*n* (%)	*n* (%)	*n* (%)	*n* (%)	*n* (%)	*n* (%)	*n* (%)	*n* (%)	*n* (%)
Radiology & nuclear medicine	42,452 (38.0)	24,042 (37.1)	18,410 (39.1)	4,353 (46.5)	8,299 (41.2)	8,034 (39.5)	9,054 (37.5)	10,366 (32.7)	2,346 (38.3)
Paediatrics	17,224 (15.4)	9,784 (15.1)	7,440 (15.8)	1,648 (17.6)	4,454 (22.1)	4,269 (21.0)	4,567 (18.9)	2,236 (7.0)	50 (0.8)
Surgery & post-surgery	10,253 (9.2)	6,263 (9.7)	3,990 (8.5)	445 (4.8)	1,183 (5.9)	1,434 (7.0)	2,724 (11.3)	3,835 (12.1)	632 (10.3)
Neurosurgery and neurology	9,605 (8.6)	5,828 (9.0)	3,777 (8.0)	1,116 (11.9)	2,477 (12.3)	1,814 (8.9)	1,729 (7.2)	2,131 (6.7)	338 (5.5)
Emergency	7,960 (7.1)	5,093 (7.9)	2,867 (6.1)	234 (2.5)	688 (3.4)	884 (4.3)	1,198 (5.0)	4,180 (13.2)	776 (12.7)
Oncological & hematopoietic diseases	5,843 (5.2)	3,374 (5.2)	2,469 (5.2)	205 (2.2)	1,078 (5.4)	1,242 (6.1)	1,233 (5.1)	1,620 (5.1)	465 (7.6)
Otorhinolaryngological diseases	5,384 (4.8)	2,954 (4.6)	2,430 (5.2)	271 (2.9)	908 (4.5)	1,304 (6.4)	1,423 (5.9)	1,339 (4.2)	139 (2.3)
Orthopaedics & traumatology	1,917 (1.7)	1,142 (1.8)	775 (1.6)	1 (0.0)	18 (0.1)	163 (0.8)	450 (1.9)	1,119 (3.5)	166 (2.7)
Unknown & no classification	1,390 (1.2)	757 (1.2)	633 (1.3)	75 (0.8)	99 (0.5)	130 (0.7)	263 (1.1)	645 (2.1)	178 (2.9)
Respiratory diseases	1,128 (1.0)	610 (0.9)	518 (1.1)	53 (0.6)	195 (1.0)	231 (1.1)	195 (0.8)	356 (1.1)	98 (1.6)
Internal medicine	1,154 (1.0)	674 (1.0)	480 (1.0)	53 (0.6)	102 (0.5)	102 (0.5)	123 (0.5)	616 (1.9)	158 (2.6)
Allergies, general medicine and epidemiology	1,080 (1.0)	624 (1.0)	456 (1.0)	58 (0.6)	119 (0.6)	193 (0.9)	151 (0.6)	458 (1.4)	101 (1.6)
Intensive medicine	1,026 (0.9)	707 (1.1)	319 (0.7)	22 (0.2)	43 (0.2)	43 (0.2)	82 (0.3)	682 (2.1)	154 (2.5)
Oral cavity	976 (0.9)	476 (0.7)	500 (1.1)	1 (0.0)	16 (0.1)	69 (0.3)	321 (1.3)	454 (1.4)	115 (1.9)
Vascular & cardiac diseases	899 (0.8)	556 (0.9)	343 (0.7)	68 (0.7)	60 (0.3)	64 (0.3)	98 (0.4)	469 (1.5)	140 (2.3)
Nursing and neonatology	786 (0.7)	455 (0.7)	331 (0.7)	645 (6.9)	26 (0.1)	26 (0.1)	18 (0.1)	57 (0.2)	14 (0.2)
Ophthalmological diseases	664 (0.6)	346 (0.5)	318 (0.7)	19 (0.2)	175 (0.9)	156 (0.8)	148 (0.6)	145 (0.5)	21 (0.3)
Digestive diseases	362 (0.3)	173 (0.3)	189 (0.4)	10 (0.1)	41 (0.2)	26 (0.1)	54 (0.2)	184 (0.6)	47 (0.8)
Genitourinary system diseases	299 (0.3)	195 (0.3)	104 (0.2)	11 (0.1)	22 (0.1)	20 (0.1)	25 (0.1)	174 (0.5)	47 (0.8)
Anaesthesiology and pain management	203 (0.2)	132 (0.2)	71 (0.2)	8 (0.1)	18 (0.1)	19 (0.1)	26 (0.1)	119 (0.4)	13 (0.2)
Psychiatry and psychology	258 (0.2)	170 (0.3)	88 (0.2)	4 (0.0)	10 (0.0)	26 (0.1)	53 (0.2)	126 (0.4)	39 (0.6)
Physical medicine & physical therapy	262 (0.2)	146 (0.2)	116 (0.2)	15 (0.2)	28 (0.1)	39 (0.2)	76 (0.3)	93 (0.3)	11 (0.2)
Endocrinology and Nutrition	169 (0.2)	55 (0.1)	114 (0.2)	5 (0.1)	10 (0.0)	18 (0.1)	37 (0.2)	82 (0.3)	17 (0.3)
Dermatology	176 (0.2)	75 (0.1)	101 (0.2)	12 (0.1)	24 (0.1)	30 (0.1)	30 (0.1)	68 (0.2)	12 (0.2)
Infectious diseases	156 (0.1)	101 (0.2)	55 (0.1)	14 (0.1)	36 (0.2)	19 (0.1)	24 (0.1)	56 (0.2)	7 (0.1)
Obstetrics and gynaecology	123 (0.1)	0 (0.0)	123 (0.3)	6 (0.1)	1 (0.0)	4 (0.0)	10 (0.0)	72 (0.2)	30 (0.5)
Rheumatology	72 (0.1)	20 (0.0)	52 (0.1)	2 (0.0)	1 (0.0)	6 (0.0)	8 (0.0)	41 (0.1)	14 (0.2)

**Fig. 3:**
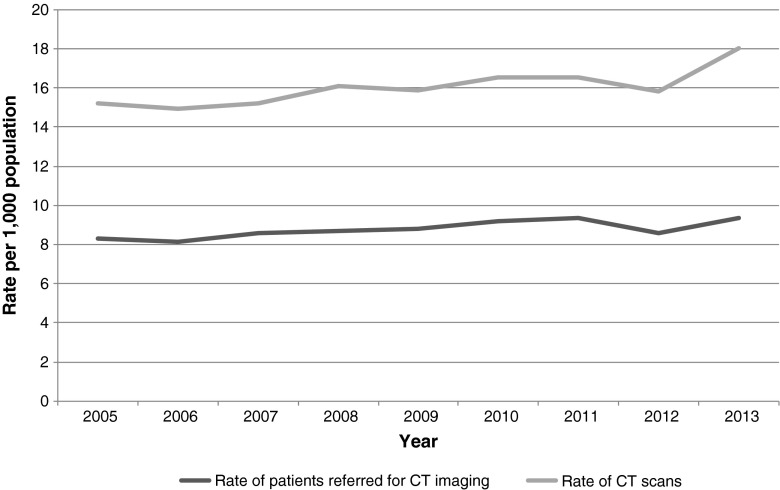
Rate of children and young adults referred for CT, and rate of CT scans per 1,000 people in the referral population (age 21 and younger) in the eight participating hospitals in Catalonia (2005–2013)

When the number of CT scans and scanned patients per year was compared to the general referral population of similar age attended at the participating hospitals, there was a non-statistically significant change from a rate of 8.3 scanned patients per 1,000 population in 2005 to 9.4 scanned patients/1,000 population in 2013 (Fig. 3). In terms of the number of CT scans per 1,000 population, the rate changed from 15.2 CT scans in 2005 to 16.6 in 2012, with a subtle increase in 2013 to 18 CT scans per 1,000 population (not statistically significant). No information on the referral population was available for earlier years.

## Discussion

Statistics on the use of CT in the National Spanish Health System have been provided by the Ministry of Health, Social Services and Equality since 1996 [2]. According to official data, the late 1990s witnessed the beginnings of an exponential use of CT imaging, with a 143% increase in the annual rate of CT scans (34.9 to 85.0 CT scans per 1,000 population) from 1996 to 2011 in Spain, 2011 being the calendar year with the latest available information [2]. In Catalonia, based on our results, a sustained increase in the number of CT scans and a less pronounced increase in the number of scanned patients were observed during the 23-year study period (1991–2013). The gradual digitalization of the radiology services among the participating hospitals, an increase in the number of scans per patient, an increase in the referral population attended at the participating hospitals, the greater number of CT scanners within the participating hospitals and the technical advances in CT equipment that allow implementation for new indications may explain the increase seen in the absolute number of CTs in the last two decades in these hospitals. It is important to emphasize that since 2004, when RIS was fully operating in all but one of the participating hospitals, an increase in the number of CT scans and scanned patients was evident though slightly less accentuated than in previous years. Therefore, the gradual RIS implementation in the hospitals may have affected the results in the number of CT scans and scanned patients in the early years, but not when most hospitals were routinely using RIS to manage and store their radiology, so we are confident that these results reliably reflect CT scan use.

The observed decrease in 2012 could be related to the increasing awareness of cancer risks following the publication of Pearce et al. [10], showing an increased risk of leukaemia and brain tumours in young people from CT scanning, which could have had an effect on the clinical practice of the radiologists at the participating hospitals. Additional data on CT scan frequency in the next years would be required to confirm this hypothesis. Most of the scanned patients received only one CT scan, although performing multiple CT scans in the same patient became increasingly common practice. Between 1991 and 2013, an increase in the frequency of patients receiving more than three CT scans per year was observed, suggesting a change in the clinical practice favouring the follow-up of diseases through CT scanning or a gradually wider use of CT scans for new indications. Although the current knowledge on enhanced radiation sensitivity of neonates and young infants is well known [14], a significant proportion of patients in the study had their first CT scans at very young ages (26.3% of the patients were younger than 5 years).

In Spain no information has been published on the trends and patterns of use of radiologic diagnostic procedures in children and young adults, hence the importance of describing the use of medium- to high-dose radiologic procedures in these patients. In the participating Catalan hospitals no increasing trend in the rate of scanned children and young adults and scans per 1,000 population was observed in the period 2005–2013, when official referral population figures were available. While the rate of CT scans and scanned patients per 1,000 population has not increased, there has been a considerable increase in the absolute number of CT scans and children and young adults referred for CT imaging in the 8-year period for which official demographic data are available, which is closely followed by a proportional increase in the population of those younger than 21 years served by the participating hospitals. Over this 8-year period, the rate of young patients scanned per 1,000 in the referral population exceeded the doubling of the rate observed in Northern England in 2002 [24] and was similar to the rate described in Australia [25] for the 5 to <15 years age range in the same period.

The availability of CT scanners has increased rapidly in most major industrialized countries over the last two decades [1], which in most cases has translated into a swift augmentation of the number of CT scans performed yearly. In the whole of Spain, the availability of CT scanners doubled from 1996 to 2011 (available data period), from 0.9 to 1.6 CT scanners per 100,000 population. In Catalonia, the number of CT scanners tripled in the same time period, from 0.6 to 1.5 scanners per 100,000 population, suggesting potentially different intensities of use in this autonomous community [2]. The observed increase in scanner availability could respond to a growing demand of this imaging equipment since their gradual introduction in the Catalan hospitals. The escalating array of diagnostic and therapeutic CT scan indications, the nature of the services provided by the participating hospitals and the increasing attending population could have determined the adoption of this diagnostic technology at the hospitals and therefore the upward trend in available scanners.

A greater use of high-dose CT examinations such as head CT (including neck examinations), thorax, and abdomen (including pelvis examinations) was observed in Catalonia and similarly in the United States [26], United Kingdom [24], and the Netherlands [27] (although in the last, “extremities” imaging exceeded the frequency of “abdomen” imaging). Although there were differences in absolute and relative frequency of imaging among children and young adults, these three anatomical regions accounted for 76% of scans in the United States and 90.9% in Northern England among CT scanning performed in children and young adults, suggesting similar uses in these two countries, as well as in Catalonia. The Netherlands study presented a closer distribution of frequency to that observed in the present study for head/neck and thorax imaging [27]. The potential decrease observed in the relative frequency of head/neck imaging over the study period could be attributed to a shift in the use of CT scans to other non-ionising diagnostic techniques. Regarding the decrease in the relative frequency of head CT scans, MRI has progressively become the gold standard for the investigation of cephalgia, especially in outpatient visits, providing wider detailed information than CT. With regard to the decrease in the relative frequency of neck CTs, the technical improvements in ultrasound have allowed its use for the investigation and follow-up of neck disorders (such as lymphadenopathies, abscesses, tumours and branchial cleft cysts) and abdomen disorders (such as Crohn disease) that previously were commonly investigated using CT imaging. On the other hand, the relative frequency of thorax CT imaging seemed to increase, potentially explained by the non-existence of a diagnostic alternative beyond conventional radiology to investigate, e.g., lung parenchyma disorders.

The rates of the referring medical specialties in Catalonia compared to other countries suggest national variation in radiologic diagnostic practices; for comparison, in Denmark the most common referring specialty was orthopaedics, followed by paediatrics and then oncology, digestive system specialities, and surgery [28], whereas the majority of requests in Catalonia were from paediatrics, surgery, and neurosurgery and neurology specialties.

It is important to bear in mind that paediatric surgery does not exist as a medical specialty in most countries excepting Catalonia. Typically, in most paediatric hospitals different surgical specialties are encompassed under a wider paediatric surgery department, possibly explaining why surgery, is among the specialties ordering most of the CT scans.

The difference in the medical specialties ordering a CT scan for very young children compared to those ordering a CT for the 15- to <20 years age group suggests different rates in specific pathologies and injuries. CTs in younger children could be related to congenital and neurodevelopmental abnormalities and also to a higher frequency of brain tumours typically seen in the youngest age groups, whereas the high rate of CT scanning in the emergency ward in adolescents and young adults could be related to a higher number of traumatic episodes, including road accidents, quarrels and suicides.

CT has been the cornerstone of imaging for more than 20 years, but orders for CT and its associated ionising radiation have to balance favourably with the diagnostic benefits. In the present study, 4.2% of patients received more than five CT scans before turning 21 years old and 1.2% received 10 or more CT scans, 41 being the maximum number of CT scans recorded in the same patient. In addition to the widely studied risks associated with the ionising radiation exposure of CT scans, it is important to take into account the economic burden associated with this procedure, especially in recession times where all public health expenditure across countries of the Organisation for Economic Co-operation and Development (OECD) have experienced cuts [1]. It is precisely this economic climate that, together with the increasing consciousness of the potential health effects of the procedure, may have played a role in modulating the high demand of these expensive medical technologies, but further analysis comparing countries with public and private health systems is needed to validate this.

## Conclusion

This study showed the patterns of use of CT imaging in patients younger than 21 years in eight major medical centres treating paediatric and young adult patients in Catalonia during a 23-year study period. The observed gradual increase in examinations and patients referred for CT imaging may be related to an increase in the availability of CT scanners within the hospitals, an increase in the population attended in the participating hospitals, an increase in the number of scans per patient and new CT scanning indications. The initial findings that showed an upward trend in the use of CT scanning in the eight participating hospitals are in agreement with previous studies on paediatric CT scan use.
